# Phospholipidation of nuclear proteins by the human papillomavirus E6 oncoprotein: implication in carcinogenesis

**DOI:** 10.18632/oncotarget.26140

**Published:** 2018-09-25

**Authors:** Benjamin Marx, Martin Hufbauer, Paola Zigrino, Slawomir Majewski, Birgid Markiefka, Timo Sachsenheimer, Britta Brügger, Baki Akgül

**Affiliations:** ^1^ Institute of Virology, University of Cologne, Cologne, Germany; ^2^ Department of Dermatology and Venereology, University Hospital Cologne, Cologne, Germany; ^3^ Department of Dermatology and Venereology, Medical University of Warsaw, Warsaw, Poland; ^4^ Institute of Pathology, University Hospital Cologne, Cologne, Germany; ^5^ Heidelberg University Biochemistry Center (BZH), Heidelberg, Germany

**Keywords:** human papillomavirus, E6 oncoprotein, phosphatidylinositides, nuclear phosphatidylinositol-4,5-bisphosphate, skin cancer

## Abstract

Phospholipids regulate numerous cellular functions and their deregulation is known to be associated with cancer development. Here, we show for the first time that expression of the E6 oncoprotein of human papillomavirus type 8 (HPV8) leads to a profound increase in nuclear phosphatidylinositol-4,5-bisphosphate (PI(4,5)P_2_) lipid levels in monolayer cultures, that led to an aberrant phospholipidation of cellular proteins. Elevated PI(4,5)P_2_ levels in organotypic skin cultures, skin tumors of K14-HPV8-E6 transgenic mice as well as HPV8 positive skin carcinomas highly suggest a decisive role of PI(4,5)P_2_ in HPV associated squamous-cell-carcinoma development. Furthermore, mass-spectrometric analysis confirmed an increase of PI(4,5)P_2_, which was characterized by a shift in the distribution of lipid species. PI(4,5)P_2_ upregulation was independent of E6 interference with MAML1. However, E6 does interfere with the PI(4,5)P_2_ metabolic pathway by upregulation of phosphatidylinositol-4-phosphate-5-kinase type I and phosphatidylinositol-5-phosphate 4-kinase type II as well as the binding to 5’-phosphatase OCRL and phosphatidylinositol. All of these mechanisms combined may contribute to PI(4,5)P_2_ elevation in E6 positive cells. The identification of CAND1 and SND1 – two proteins known to be involved in carcinogenic processes – were significantly stronger phospholipidized in the presence of E6. In conclusion we provide evidence that the modulation of the PI(4,5)P_2_ metabolism is a novel oncogenic mechanism relevant for HPV-induced carcinogenesis.

## INTRODUCTION

Human papillomaviruses (HPVs) are DNA viruses infecting both mucosa and skin, where they can persist asymptomatically or cause keratinocyte cancer. High-risk HPVs of genus alphapapillomavirus (alphaPV, e.g. HPV16) are the established cause of cervical intraepithelial neoplasia (CIN) and their progression to cervical cancer [1]. Also, epidemiological as well as experimental data support a carcinogenic role of HPV of genus betapapillomavirus (betaPV, e.g. HPV8) in the development of squamous cell skin carcinoma (SCC), especially in high-risk patient groups such as immunosuppressed organ transplant recipients [2–4]. Initial evidence for a role of cutaneous HPVs in the pathogenesis of skin malignancies arose from the identification of HPV5 and HPV8 in patients suffering from the rare genetic disorder Epidermodysplasia verruciformis (EV). EV is characterized by diffuse, wart-like lesions with broad areas of the skin being frequently transformed into carcinomas early in life [5]. Functional studies on betaPV early gene products showed that the viral E6 oncoprotein inhibits apoptosis and DNA damage repair following UV irradiation [6, 7]. In addition to the disruption of the cellular UV response, E6 also has the capacity to bind the Mastermind-like protein 1 (MAML1), thus inhibiting NOTCH-dependent keratinocyte differentiation [8–10]. Moreover, the ability of E6 to bind MAML1 has proven to be a required prerequisite for skin papilloma formation in mouse papillomavirus type 1 infected mice [11]. When expressed in transgenic mice under the control of the keratin-14 promoter (K14-HPV8-E6), HPV8-E6 induces the development of papillomas - partially with moderate to severe dysplasia and SCC. A single UVA/B treatment of the skin induced papillomatosis in K14-HPV8-E6 mice within 3 weeks [12]. The persistence of UV damaged cells in these mice was identified as crucial for skin tumorigenesis [13].

The E6 proteins of high-risk alphaPV are characterized by the presence of a PDZ-binding motif (PSD95-DLG1-ZO-1) through which they interact with a number of cellular PDZ domain-containing substrates and cooperate in their degradation. The ability of these E6 proteins to bind to PDZ domain proteins correlates with the oncogenic potential of the virus [14, 15]. Since the E6 proteins of oncogenic betaPV do not encode a PDZ-binding motif no cellular PDZ protein is known to be targeted for degradation. However, we recently identified the PDZ domain protein Syntenin-2 to be transcriptionally downregulated by E6, but not by E7 of HPV8 [16]. Syntenin-2 is known to interact with high affinity with phosphatidylinositol-4-5-bisphosphate (PI(4,5)P_2_) [17]. Recent studies have implied an association of phosphoinositides and their metabolizing enzymes with various pathophysiological conditions, including cancer [18]. PI(4,5)P_2_ is a minor lipid that binds and regulates the activity of various proteins. Nuclear PI(4,5)P_2_ is regulated independently of the cytoplasmic phosphoinositide pool [19], and has been localized to nucleoli and nuclear speckles (also known as interchromatin granule clusters) [20, 21], which are nuclear domains enriched in pre-mRNA splicing factors, located in the interchromatin regions of the nucleoplasm of mammalian cells [22]. Nuclear PI(4,5)P_2_ levels are altered in response to numerous cellular stimuli, strongly supporting the notion of PI(4,5)P_2_ acting as a direct messenger that binds and regulates nuclear effectors [19, 23, 24]. Recent studies have revealed that nuclear PI(4,5)P_2_ can directly bind and regulate functions of nuclear proteins involved in multiple processes, including transcription, mRNA processing and export, chromatin remodeling, stress responses, DNA repair as well as mitosis (reviewed in [25, 26]). However, the exact role of nuclear PI(4,5)P_2_ in epidermal cancer development still needs to be resolved [21, 27]. Yet, in this study we now demonstrate for the first time that HPV targets the PI(4,5)P_2_ metabolic pathway, leading to enhanced phospholipidation of nuclear proteins in skin keratinocytes. Taken together, our experimental results provide evidence for a novel oncogenic mechanism relevant for HPV induced carcinogenic processes in keratinocytes.

## RESULTS

### Expression of HPV8-E6 increases nuclear PI(4,5)P_2_

**Figure 1:**
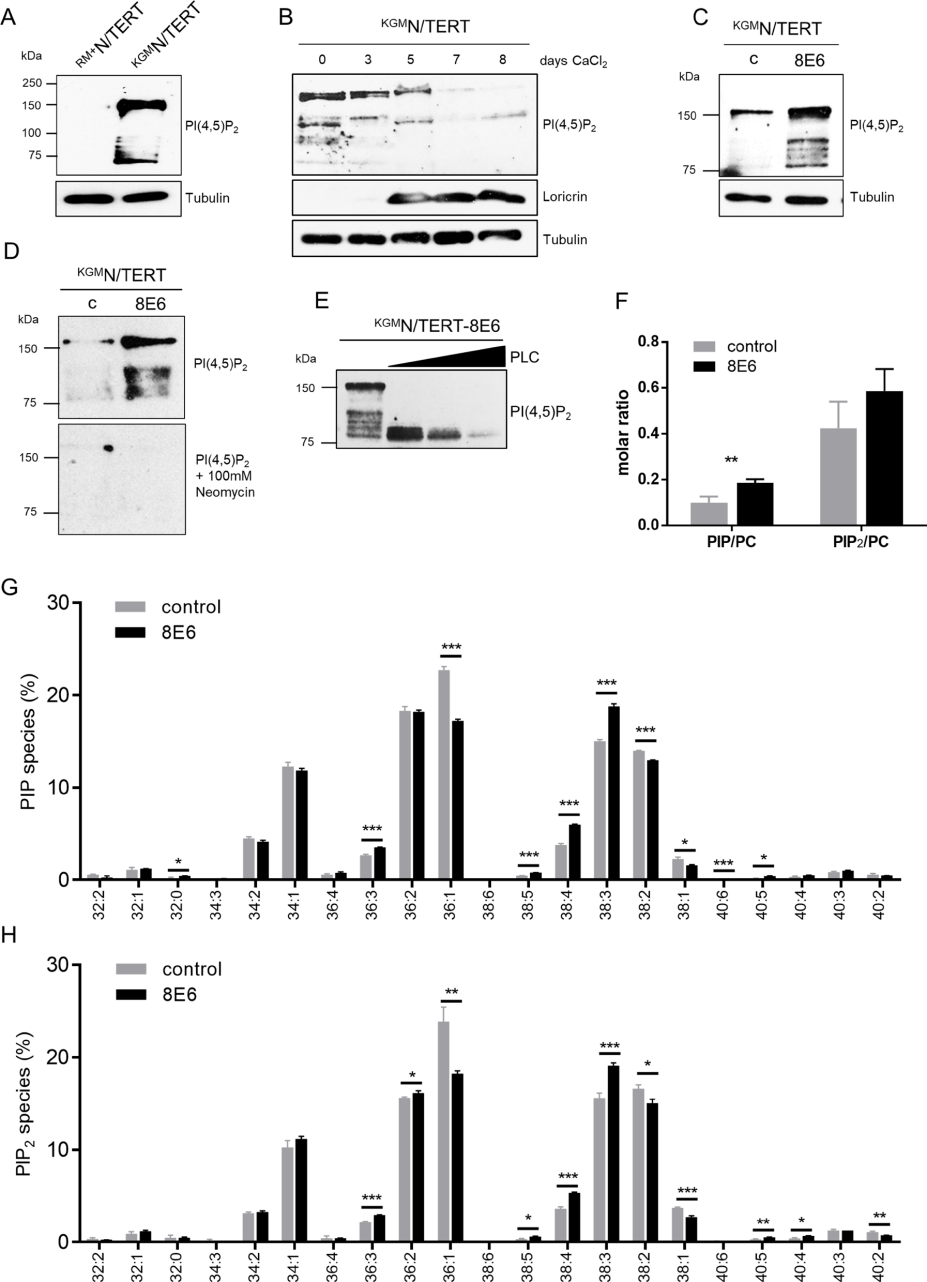
Expression of HPV8-E6 leads to high PI(4,5)P_2_ levels in N/TERTs cultured in low calcium media (**A**) Representative Western blot for PI(4,5)P_2_ in total cell extracts of ^RM+^N/TERTs and ^KGM^N/TERTs. Equal protein loading was confirmed by immunoblotting for Tubulin. (**B**) Representative Western blot for PI(4,5)P_2_ in total cell extracts of ^KGM^N/TERT grown in low calcium and then switched to high calcium concentrations for up to 8 days. Calcium induced keratinocyte differentiation was confirmed by Western blotting for Loricrin. Equal protein loading was confirmed by immunoblotting for tubulin. (**C**) Representative Western blot for PI(4,5)P_2_ in total cell extracts of empty vector positive ^KGM^N/TERT-pLXSN or ^KGM^N/TERT-8E6. Difference in intensity of PI(4,5)P_2_ bands in ^KGM^N/TERTs between Figure 1A and 1D is due to 80 μg total protein and long exposure of blot to film versus 40 μg total protein and short exposure. Equal protein loading was confirmed by immunoblotting for tubulin. (**D**) Representative Western blot for PI(4,5)P_2_ in total cell extracts of ^KGM^N/TERT-pLXSN and ^KGM^N/TERT-8E6 treated with skimmed milk containing either PI(4,5)P_2_ specific antibodies (top) or in milk containing100 mM Neomycin (bottom). (**E**) Total cell extracts of ^KGM^N/TERT-8E6 were treated with 0, 5, 10 or 20 mU Phospholipase C (PLC). (**F**) Quantification of phosphoinositide levels in HPV8-E6 expressing keratinocytes. Relative amounts of PIP and PIP_2_ levels in ^KGM^N/TERT-pLXSN and ^KGM^N/TERT-8E6 cells. Bars present phosphoinositide values normalized to phosphatidylcholine (PC) levels (PIP_x_/PC). Data are presented as mean ± SD (*n =* 3). (**G**–**H**) Effect of E6 expression on the relative distribution of PIP (**G**) and PIP_2_ (**H**) molecular species. Lipid species are annotated based on their fatty acyl composition x:y, with x = total number of C atoms in both fatty acyl chains, and y = total number of double bonds in both fatty acyl chains). Data are normalized to 100% within each lipid class and are displayed as mean ± SD (*n =* 3).

To test whether PI(4,5)P_2_ is detectable in skin keratinocytes, total cell extracts of N/TERT cells (N/TERTs) grown either in KGM-Gold (low calcium media; ^KGM^N/TERTs) or in RM^+^ media (high calcium media; ^RM+^N/TERTs) were generated [28]. This cell line derived from foreskin keratinocytes and was immortalized through overexpression of the catalytic subunit of human telomerase [29]. Western blots were performed using the mouse monoclonal anti-PI(4,5)P_2_-IgM antibody that specifically detects PI(4,5)P_2_ [30]. Surprisingly, despite the fact that PI(4,5)P_2_ is a very tiny molecule and should therefore only have been detectable at the dye front in SDS-PAGEs, the PI(4,5)P_2_ specific antibody unexpectedly recognized multiple protein bands in the range of 75–150 kDa in ^KGM^N/TERTs, which were absent in ^RM+^N/TERTs (Figure 1A). This observation implies that ^KGM^N/TERTs with basal cell characteristics may contain proteins bound to PI(4,5)P_2_. To address the question whether PI(4,5)P_2_ signals might be dependent on keratinocyte differentiation, ^KGM^N/TERTs were treated with extracellular calcium (2 mM) for up to 8 days. As shown in Figure 1B, Loricrin (a marker for terminal keratinocyte differentiation) was expressed 5 days following calcium exposure. Furthermore, keratinocyte differentiation was paralleled by a decrease of PI(4,5)P_2_-bound protein bands. Most intriguingly, the expression of HPV8-E6 in ^KGM^N/TERTs (^KGM^N/TERT-8E6) led to significantly stronger PI(4,5)P_2_-bound protein band signals when compared to matched control (Figure 1C).

The specificity of the anti-PI(4,5)P_2_ antibody (clone 2C11) has been proven in other studies before [30, 31]. Pre-incubation of the 2C11 antibody with an excess of liposomes containing different phosphoinositides showed that this lipid oversaturation led to an abrogation of specific staining of PI(4,5)P_2_ [30, 31]. Furthermore, in another study it was shown that PI(4,5)P_2_ binding of 2C11 was effectively prevented by neomycin, an aminoglycoside antibiotic that binds with high affinity to several phosphoinositides [21]. To further confirm that the observed Western blot signals were PI(4,5)P_2_ specific, we also conducted experiments to confirm the specificity of 2C11 for PI(4,5)P_2_. To this end, we added neomycin while blocking the membranes, and also observed an absence of protein bands compared to untreated blots (Figure 1D). Additionally, PI(4,5)P_2_ signals in Western blots also disappeared following pre-incubation of cell extracts with phospholipase C, an enzyme that hydrolyzes PI(4,5)P_2_ to diacylglycerol and inositol-(1,4,5)-triphosphate. All these experiments further underpinned the specificity of the antibody signal (Figure 1E).

Mass-spectrometric analysis of phosphoinositide species showed an increase of both mono- and bis-phosphorylated phosphoinositides in HPV8-E6 expressing N/TERTs (Figure 1F). The increase of PI(4,5)P_2_ was accompanied by a shift in lipid species distribution identified by mass-spectrometric analysis. In the presence of HPV8-E6 expression polyunsaturated PIP and PIP_2_ lipid species 38:4 and 38:3, mainly comprised of the fatty acyl combinations 18:0/20:4 for lipid species 38:4 and 18:0/20:3 for lipid species 38:3, were both significantly elevated, predominantly at the expense of monounsaturated phosphoinositide depletion (Figure 1G–1H). Taken together, our data strongly suggest that HPV8-E6 enforces a basal cell-like keratinocyte phenotype, characterized by PI(4,5)P_2_ enrichment.

### High nuclear PI(4,5)P_2_ levels in HPV8-E6 positive keratinocytes and transgenic murine skin

**Figure 2:**
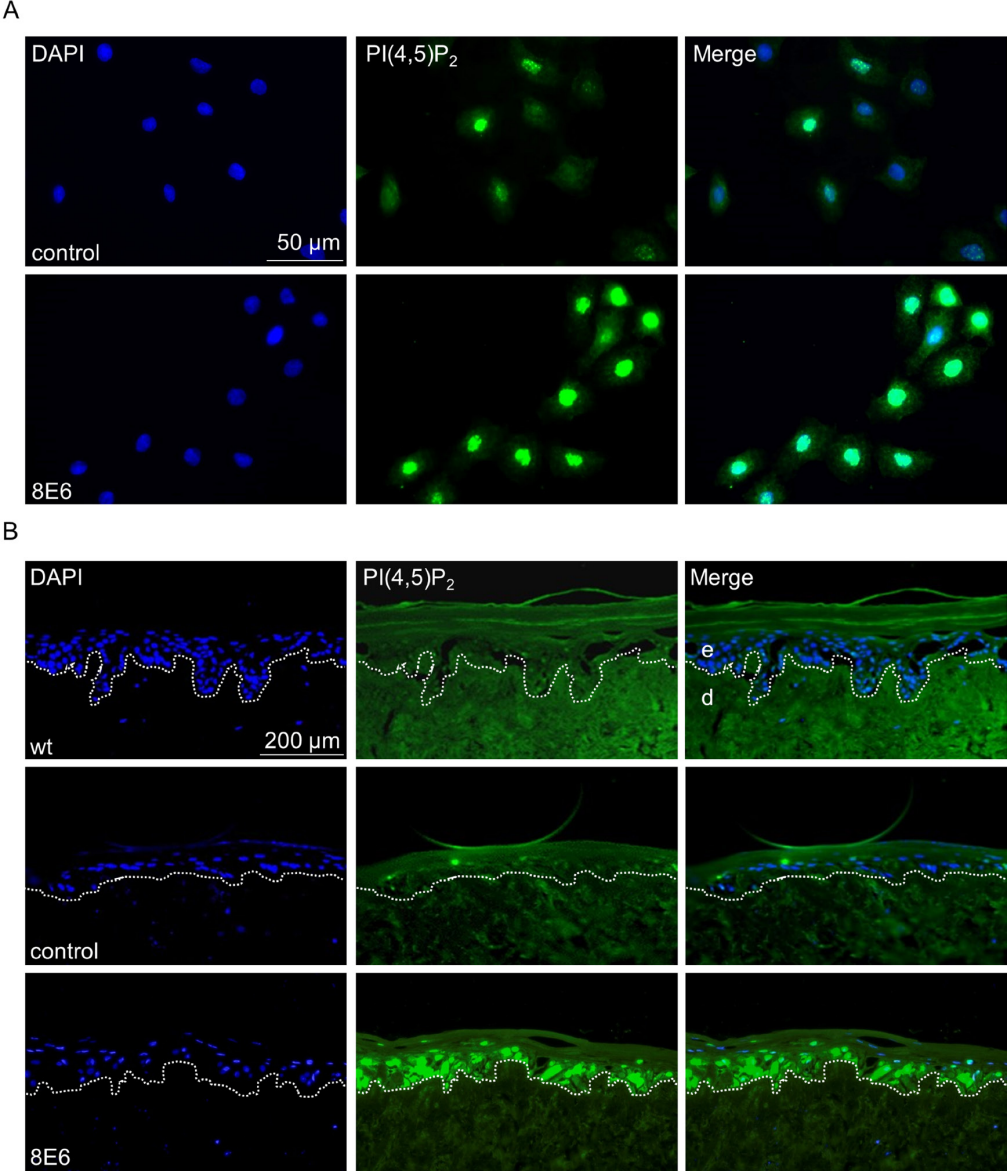
The nuclear PI(4,5)P_2_ pool is enriched in HPV8-E6 positive keratinocytes (**A**) Representative immunocytochemical staining of PI(4,5)P_2_ in ^KGM^N/TERT-pLXSN and ^KGM^N/TERT-8E6 showing an increase in nuclear PI(4,5)P_2_ levels in HPV8-E6 positive cells (blue: DAPI; green: PI(4,5)P_2_). (**B**) Representative immunofluorescence staining image of PI(4,5)P_2_ on organotypic skin cultures based on de-epidermalized human dermis as matrix, which was repopulated with wt PHK or PHK coding for the empty retroviral vector pLXSN or pLXSN-8E6. The organotypic cultures were grown for 14 days at the air-liquid interphase, followed by fixing and embedding in paraffin.

In addition to Western blot results, immunofluorescence staining also clearly showed a significant PI(4,5)P_2_ upregulation, mainly located in the nucleus in ^KGM^N/TERT-8E6 cells cultured as monolayers (Figure 2A). In organotypic skin cultures of primary human keratinocytes (PHK) only weak PI(4,5)P_2_ specific signals were detected in cultures of wild-type (wt) keratinocytes, or keratinocytes expressing the empty retroviral vector pLXSN. In contrast, strong PI(4,5)P_2_ fluorescence signals were detected in E6 positive keratinocytes throughout the regenerated epithelial keratinocyte layers (Figure 2B).

**Figure 3:**
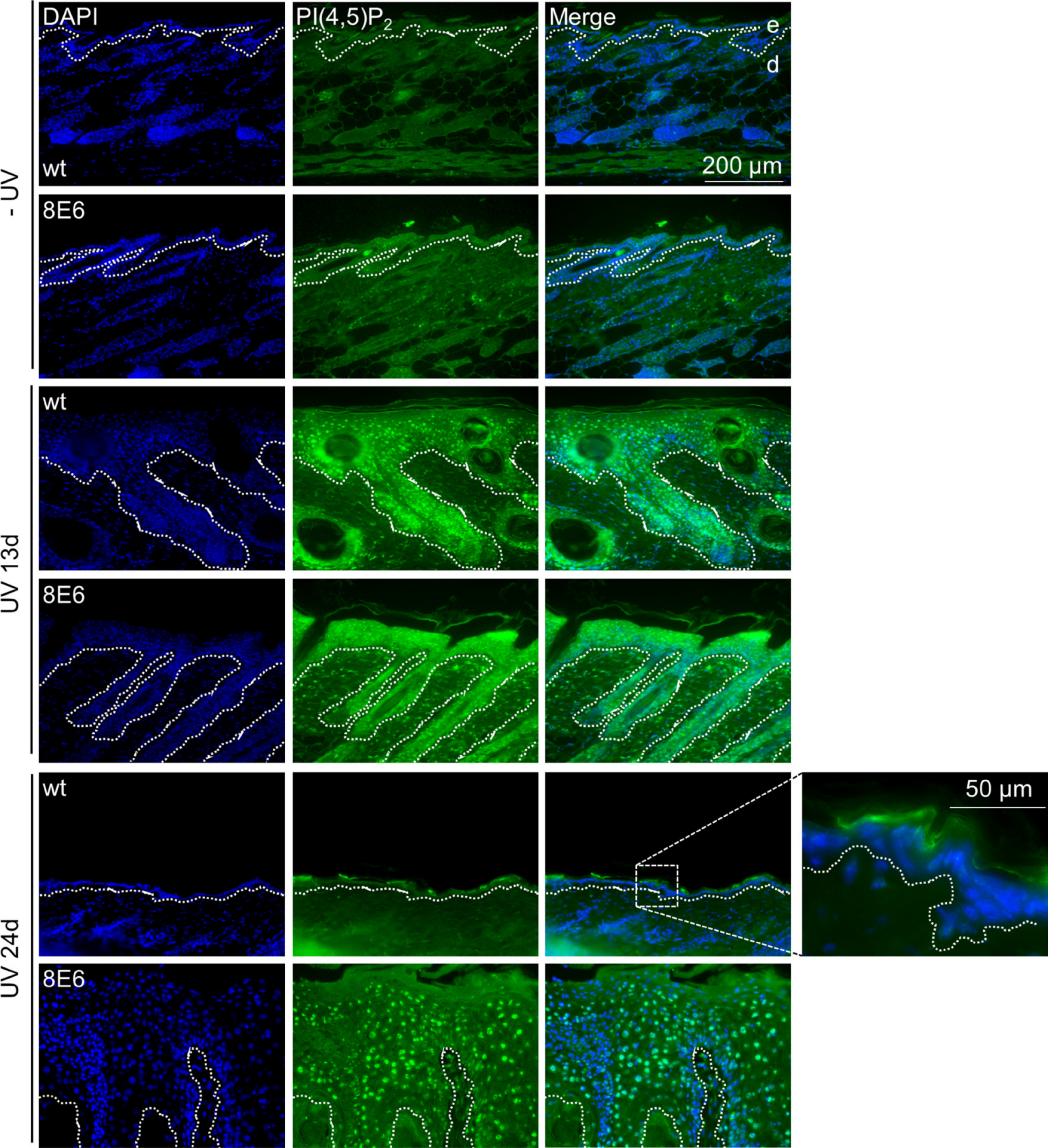
Skin tumors of K14-HPV8-E6 transgenic mice display elevated levels of PI(4,5)P_2_ Skin biopsies were taken from FVB/n-wt (*n =* 4) and K14-HPV8-E6 (*n =* 4) mice out of the UV irradiated skin area 13 days or 24 days following UV treatment. Additionally, skin biopsies were taken from non-irradiated (*n =* 4) FVB/n-wt and K14-HPV8-E6 (*n =* 4) mice, respectively. Paraffin sections were stained for PI(4,5)P_2_ (blue: DAPI; green: PI(4,5)P_2_; dashed line: basement membrane zone; d: dermis; e: epidermis). The magnified image of wt skin 24 days following UV treatment demonstrates unspecific staining in the cornified cell layer.

In addition to organotypic cultures, PI(4,5)P_2_ levels were also evaluated in untreated and UVA/B-irradiated FVB/n-wt as well as K14-HPV8-E6 transgenic murine skin. No PI(4,5)P_2_ specific signals were found in the untreated FVB/n-wt epidermis, whereas isolated signals could be detected in untreated K14-HPV8-E6 skin. Thirteen days post UV-exposure, both mouse strains developed skin hyperplasia, which was paralleled by high nuclear PI(4,5)P_2_ levels in epithelial cell layers. Twenty-four days post treatment, FVB/n-wt skin had fully healed from UV induced hyperplasia and did not show any signs of nuclear PI(4,5)P_2_ anymore, whereas - in K14-HPV8-E6 mice hyperplasia led to skin tumor formation - with persistent high levels of PI(4,5)P_2_ in the nucleus throughout the epithelium (Figure 3).

### High PI(4,5)P_2_ levels in HPV positive clinical samples

**Figure 4:**
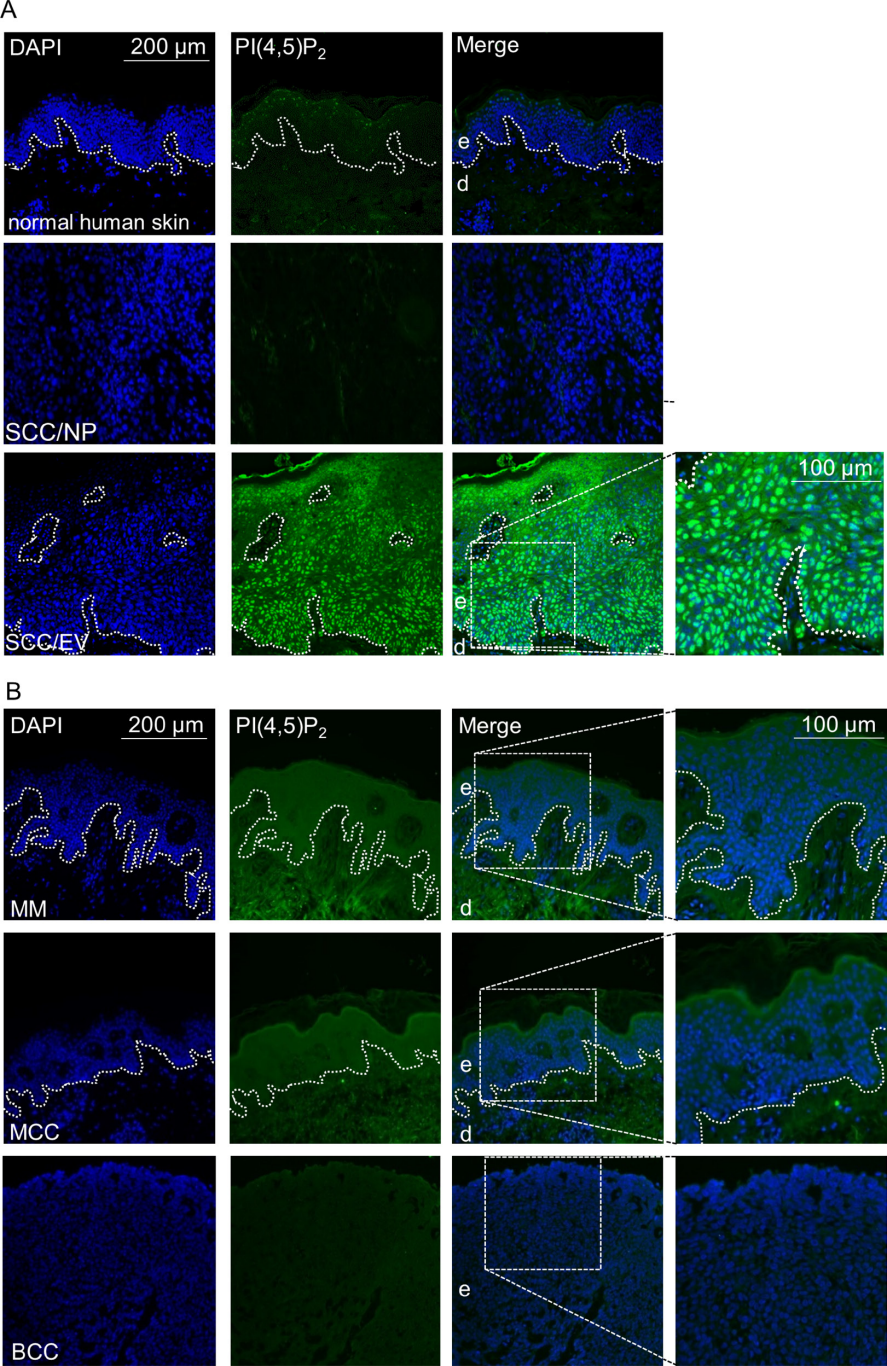
High nuclear PI(4,5)P_2_ levels in human keratinocyte skin cancer (**A**) Representative immunofluorescence staining for PI(4,5)P_2_ in normal human skin, skin SCC of the normal population (SCC/NP) and HPV8 positive EV-SCC. Histology of the EV lesions is shown by HE staining (blue: DAPI; green: PI(4,5)P_2_; dashed line: basement-membrane zone; d: dermis; e: epidermis). (**B**) Representative immunofluorescence staining for PI(4,5)P_2_ in malignant melanoma (MM, *n =* 5), Merkel cell polyomavirus positive Merkel cell carcinoma (MCC, *n =* 5) and basal cell carcinoma (BCC, *n* = 5) (blue: DAPI; green: PI(4,5)P_2_; dashed line: basement-membrane zone; d: dermis; e: epidermis).

**Figure 5:**
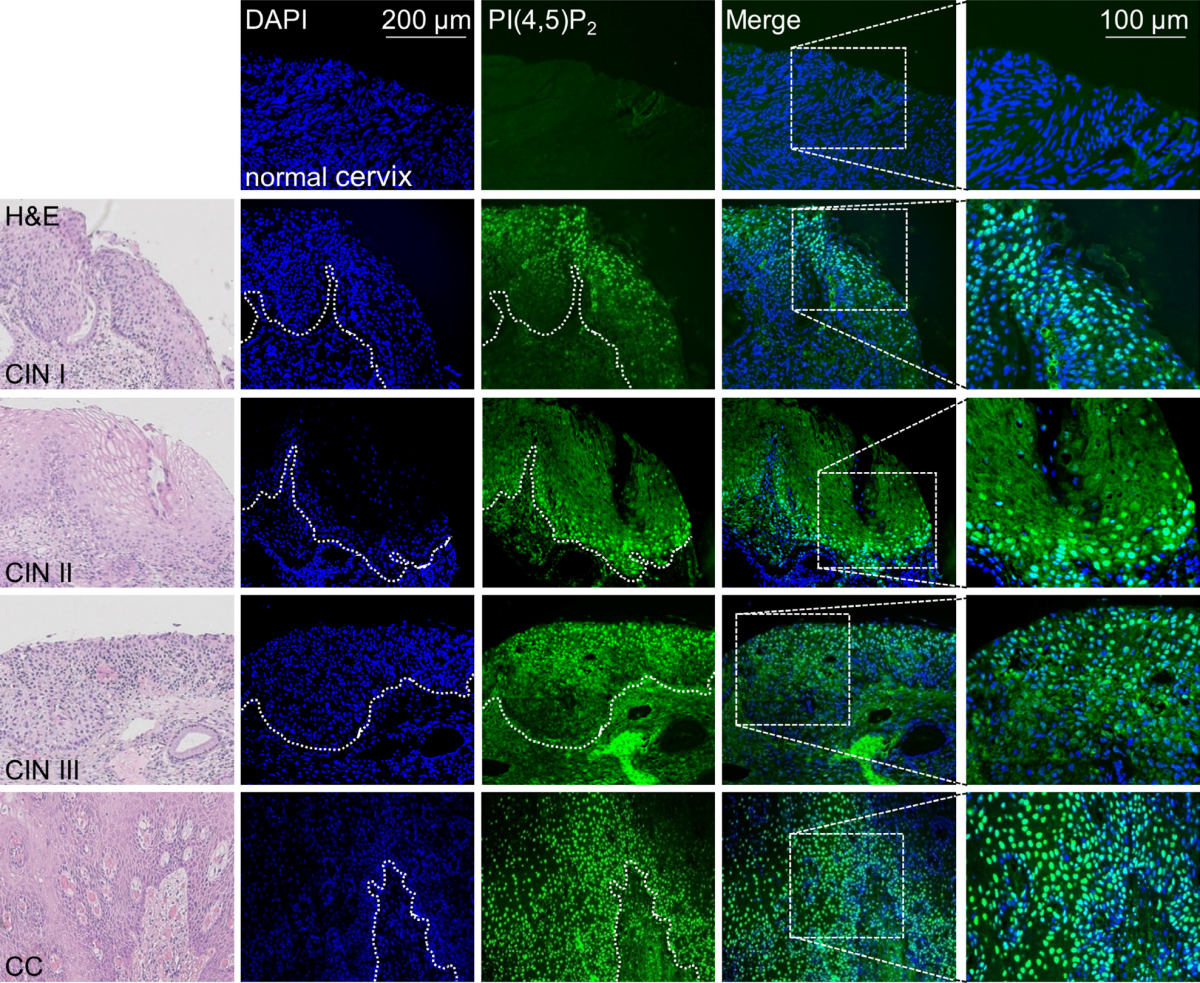
Enhanced nuclear PI(4,5)P_2_ levels in HPV16 positive cervical tumors Representative immunofluorescence staining for PI(4,5)P_2_ in normal cervix (*n =* 3), and HPV16 positive CIN I (*n =* 3), CIN II (*n =* 5), CIN III (*n =* 11) and cervical cancer (CC, *n =* 3) (blue: DAPI; green: PI(4,5)P_2_). Histology of the tissue is shown in the HE staining images.

Having demonstrated, that nuclear PI(4,5)P_2_ levels rise upon HPV8-E6 expression, PI(4,5)P_2_ levels were next studied in normal human skin as well as in SCCs of the general population, generally associated with low viral load, and in lesions of EV-patients, characterized by productive HPV infection and high viral loads. While no staining was found in healthy human skin or SCCs with low viral loads, strong nuclear localization of PI(4,5)P_2_ was detected throughout the HPV8 positive EV-tumor (Figure 4A). To exclude that the observed high PI(4,5)P_2_ levels might not be associated with other skin cancer types, such as melanoma, basal cell carcinoma, or Merkel cell polyomavirus positive Merkel cell carcinomas, these cancers were further assessed by performing immunofluorescence staining. As none of the additionally tested three skin cancer types had shown any signal of PI(4,5)P_2_ (Figure 4B), the increase of PI(4,5)P_2_ is specific for betaPV-induced skin carcinogenesis. To further evaluate whether HPV16 may also cause PI(4,5)P_2_ enrichment, we next analyzed PI(4,5)P_2_ levels in tissue-micro-arrays of HPV16 positive cervical intraepithelial neoplasia (CIN) and cervical cancers. By monitoring HPV16 gene expression in low-grade CIN and cervical cancers, Böhm *et al.* already demonstrated several years ago that in low-grade lesions E6 transcripts were only detected in the upper third of the epithelium with no significant differences between the viral transcription pattern in high-grade CIN and invasive carcinoma [32]. In healthy cervical epithelium, no PI(4,5)P_2_ signals were detected. However, there were markedly increased PI(4,5)P_2_ signals in the upper most layers of CIN I, in the basal layers of CIN II, throughout the epithelium in CIN III and cervical cancer tissue (Figure 5). The staining pattern of nuclear PI(4,5)P_2_ is therefore in accordance with E6 expression in cervical tissues. To summarize, elevated PI(4,5)P_2_ levels are a common phenomenon in oncogenic alphaPV and betaPV infected keratinocytic lesions.

### HPV8-E6 mediated enrichment of PI(4,5)P_2_ is independent from deregulated NOTCH effects

**Figure 6:**
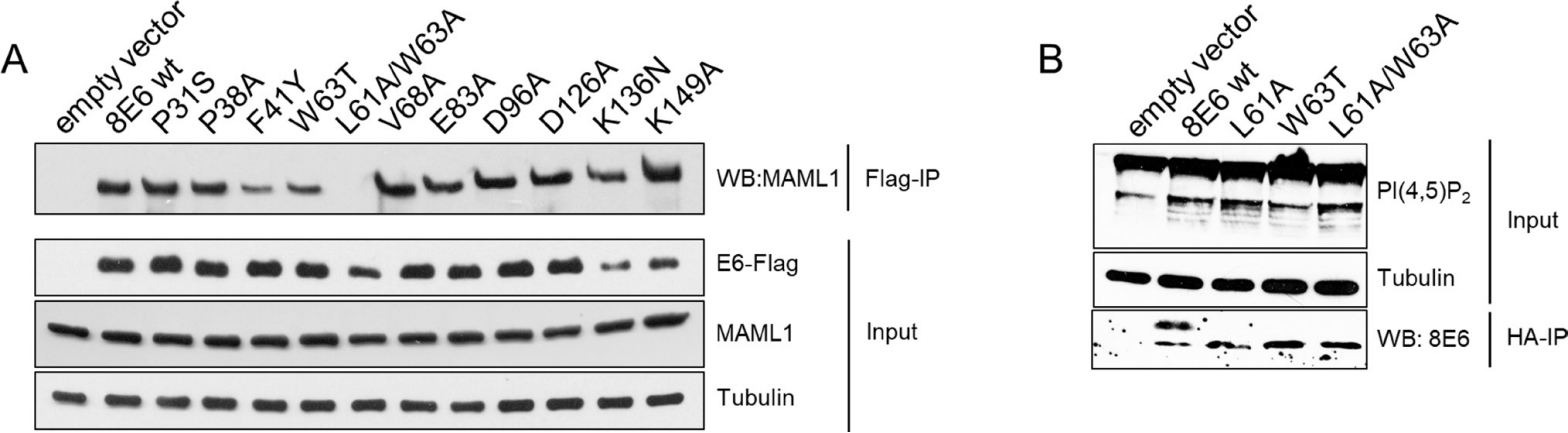
Increase of PI(4,5)P_2_ in E6 cells is independent of the E6 capability to bind to MAML1 (**A**) Extracts from C33a cells, transiently transfected with either empty vectors or plasmids coding for Flag-8E6-wt, -P31S, -P38S, -F41Y, -W63T, -L61A/W63A, -V68A, -E83A, -D96A, -D126A, -K136N, -K149A were incubated with M2-FLAG-agarose. Co-immunoprecipitated of MAML1 and 10% of the input extracts were subjected to Western blots with specific antibodies. Expression of HPV8-E6 was confirmed by a Western blot for the Flag-tagged HPV8-E6 protein. Equal protein loading was confirmed by immunoblotting for tubulin. (**B**) N/TERTs were transduced with retroviruses encoding for either the empty vector pLXSN-HA or HA-tagged HPV8-E6wt, -L61A, -W63T or L61A/W63A. PI(4,5)P_2_ was detected with specific antibodies. Expression of HPV8-E6 was confirmed by Co-IP of the HA-tagged proteins. Equal protein loading was confirmed by immunoblotting for tubulin.

The betaPV E6 proteins can bind to MAML1, a transcriptional co-activator of NOTCH regulated genes. Binding of E6 to MAML1 blocks NOTCH-dependent keratinocyte differentiation [8–10]. To determine whether the increase of PI(4,5)P_2_ may not simply result from E6-mediated disruption of the normal keratinocyte differentiation pathway, originating from interruption of the NOTCH signaling pathway, a series of HPV8-E6 mutants was generated with mutations altering conserved residues in betaPV E6. All mutant proteins were found to be stably expressed in transiently transfected C33a cells. Flag-tagged HPV8-E6wt and all other single mutants were able to bind MAML1. Interestingly, the double mutant L61A/W63A completely lost the ability to complex with MAML1 (Figure 6A). To analyze, whether the absent binding to MAML1 might result in PI(4,5)P_2_ upregulation, the single mutants L61A and W63A as well as the double mutant L61A/W63A were sub-cloned into the retroviral vector pLXSN, and recombinant retroviruses were used to transduce ^KGM^N/TERTs. As shown in Figure 6B, ^KGM^N/TERTs expressing the mutated proteins all showed elevated PI(4,5)P_2_, thus providing evidence that targeting PI(4,5)P_2_ is a specific strategy independent of HPV8-E6 disruption of NOTCH-mediated signaling.

### PI(4,5)P_2_ metabolism is targeted by HPV8-E6

**Figure 7:**
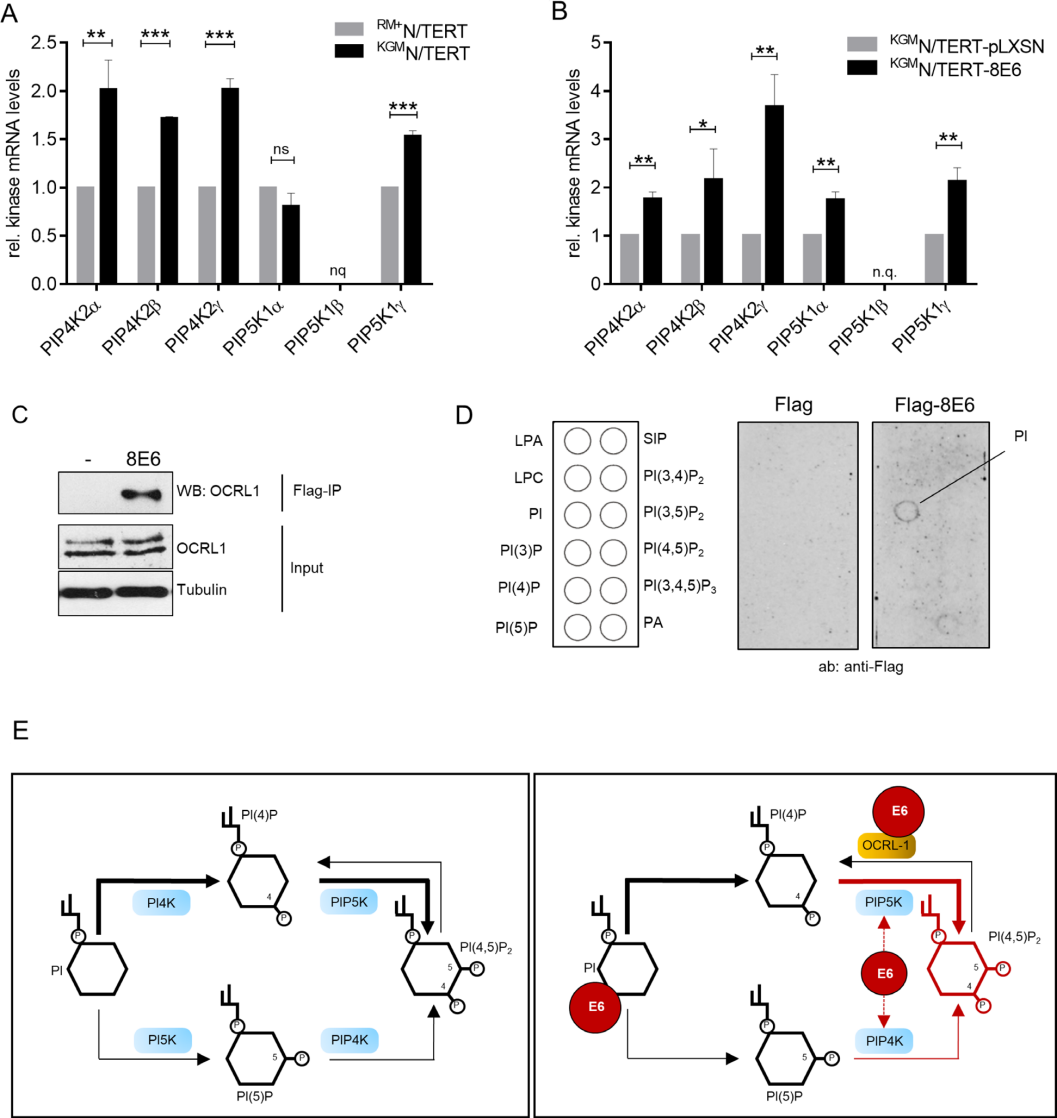
HPV8-E6 interferes with kinases and the phosphatase regulating PI(4,5)P_2_ levels (**A**) Quantification of PIP4KIIα, β, γ and PIP5KIα, β, γ mRNA expression by RT-qPCR in ^KGM^N/TERTs and ^RM+^N/TERTs (*n =* 3 independent experiments measured in duplicates). Data were normalized to the HPRT1 mRNA levels and are presented as mean ± SEM. The relative gene expression level of ^RM+^N/TERTs was set as 1 (^**^p < 0.01; ^***^p < 0.001; nq: not quantifiable; ns: not significant). (**B**) Quantification of PIP4KIIα, β, γ and PIP5KIα, β, γ mRNA expression by RT-qPCR in ^KGM^N/TERT-pLXSN and ^KGM^N/TERT-8E6 (*n =* 3 independent experiments measured in duplicates). Data were normalized to HPRT1 mRNA levels and are presented as mean ± SEM. The relative gene expression level of ^KGM^N/TERT-pLXSN was set as 1 (^*^p < 0.05; ^**^p < 0.01; nq: not quantifiable). (**C**) Extracts from C33a cells, transiently transfected with expression vectors for empty vector or Flag-HPV8-E6wt were incubated with M2-FLAG-agarose. Co-immunoprecipitated OCRL-1 and 10% of the input extracts were detected by Western blot with specific antibodies (*n =* 5). The expression of HPV8-E6 was confirmed by a Western blot against the Flag tag. Equal protein loading was confirmed by immunoblotting for tubulin. (**D**) Schematic diagram of the nitrocellulose membrane with immobilized phospholipids in 100 picomole spots (PIP-strip; left image); LPA: lysophosphatidic acid; LPC: lysophosphocholine; PI: phosphatidylinositol; SIP: sphingosine-1-phosphate; PA: phosphatidic acid. Representative images of PIP-strips incubated with total cell extracts of C33a cells transiently transfected with pXJ41-Flag (middle image) or pXJ41-8E6-Flag (right image). Membranes were washed and lipid-bound Flag-8E6 was detected with monoclonal anti-Flag antibody. (**E**) The left image shows the PI(4,5)P_2_ pathway flow in a normal keratinocyte. The right image schematically summarizes HPV8-E6 targets involved in PI(4,5)P_2_ metabolism. The figure also shows that the PIP5K pathway is the major and PIP4K the minor route of PI(4,5)P_2_ synthesis.

In principle, PI(4,5)P_2_ is synthesized by either phosphorylation of phosphatidylinositol-4-phosphate (PI4P) by the lipid kinase phosphatidylinositol-4-phosphate-5-kinase type I (PIP5KI) or by phosphorylation of phosphatidyl-inositol-5-phosphate (PI5P) by phosphatidyl-inositol-5-phosphate 4-kinase type II (PIP4KII) [33]. Each of these kinases has three isoforms, designated α, β and γ. Since the cellular levels of PI4P are much higher than those of PI5P, it is accepted that the majority of nuclear PI(4,5)P_2_ is synthesized through PIP5KI [34]. To identify the kinase isoforms involved in PI(4,5)P_2_ generation in N/TERTs, we performed RT-qPCRs, and quantified the mRNA levels of all six kinases in ^KGM^N/TERTs and ^RM+^N/TERTs. ^KGM^N/TERTs contained higher transcript levels for PIP4KIIα (2-fold, *p* = 0.0078), PIP4KIIβ (1.7, *p* < 0.0001), PIP4KIIγ (2-fold, *p* = 0.0004), and PIP5KIγ (1.5-fold, *p* = 0.0003). PIP5KIα did not show a significant difference in mRNA expression (Figure 7A). Expression of HPV8-E6 led to a significant upregulation of PIP4KIIα of about 1.7-fold (*p* = 0,0026), a 2.2-fold upregulation of PIP4KIIβ (*p* = 0,0356), a 3.7-fold upregulation of PIP4KIIγ (*p* = 0,0049), a 1,7-fold upregulation of PIP5KIα (*p* = 0,0037) and a 2-fold upregulation of PIP5KIγ (*p* = 0,0026) (Figure 7B). To identify the kinase isoform mainly contributing to the synthesis of PI(4,5)P_2_, PIP4KIIα, PIP4KIIβ, PIP5KIα and PIP5KIγ were individually silenced by using siRNA pools. Although a strong knock-down could be achieved for all four tested kinases, no significant changes of PI(4,5)P_2_ bound protein bands were observed in Western blots (Supplementary Figure 1).

With the aim to determine whether HPV8-E6 may directly interact with intracellular proteins involved in PI(4,5)P_2_ regulation, we next performed immunoprecipitation-coupled mass spectroscopy and thus identified the inositol-polyphosphate-5-phosphatase (OCRL1) as a potential interaction partner of E6 which is known to have substrate specificity for PI(4,5)P_2_ and PI(3,4,5)P_3_ [35]. The subsequent Co-IP analysis confirmed OCRL1 as a newly identified HPV8-E6 interacting phosphatase (Figure 7C).

Additionally, we addressed the question whether there may be direct phospholipid-binding of E6. We therefore transfected keratinocytes with a plasmid encoding for a Flag-HPV8-E6 fusion protein and subsequently generated total cell extracts and used these extracts to test the E6 binding capacity to phospholipids in a protein-lipid overlay assay on PIP-Strips. Intriguingly, this overlay assay, developed with an anti-Flag antibody, revealed binding specificity of E6 to phosphatidylinositol (PI), whereas cross-reactivity with various phospholipids, including PI(4,5)P_2_ and other phosphoinositides was negligibly small (Figure 7D).

In summary, as schematically outlined in Figure 7E, our results provide new mechanistic insights identifying the PI(4,5)P_2_ metabolic pathway as new target of HPV8-E6.

### CAND1 and SND1 are phospholipidized in HPV8-E6 positive keratinocytes

**Figure 8:**
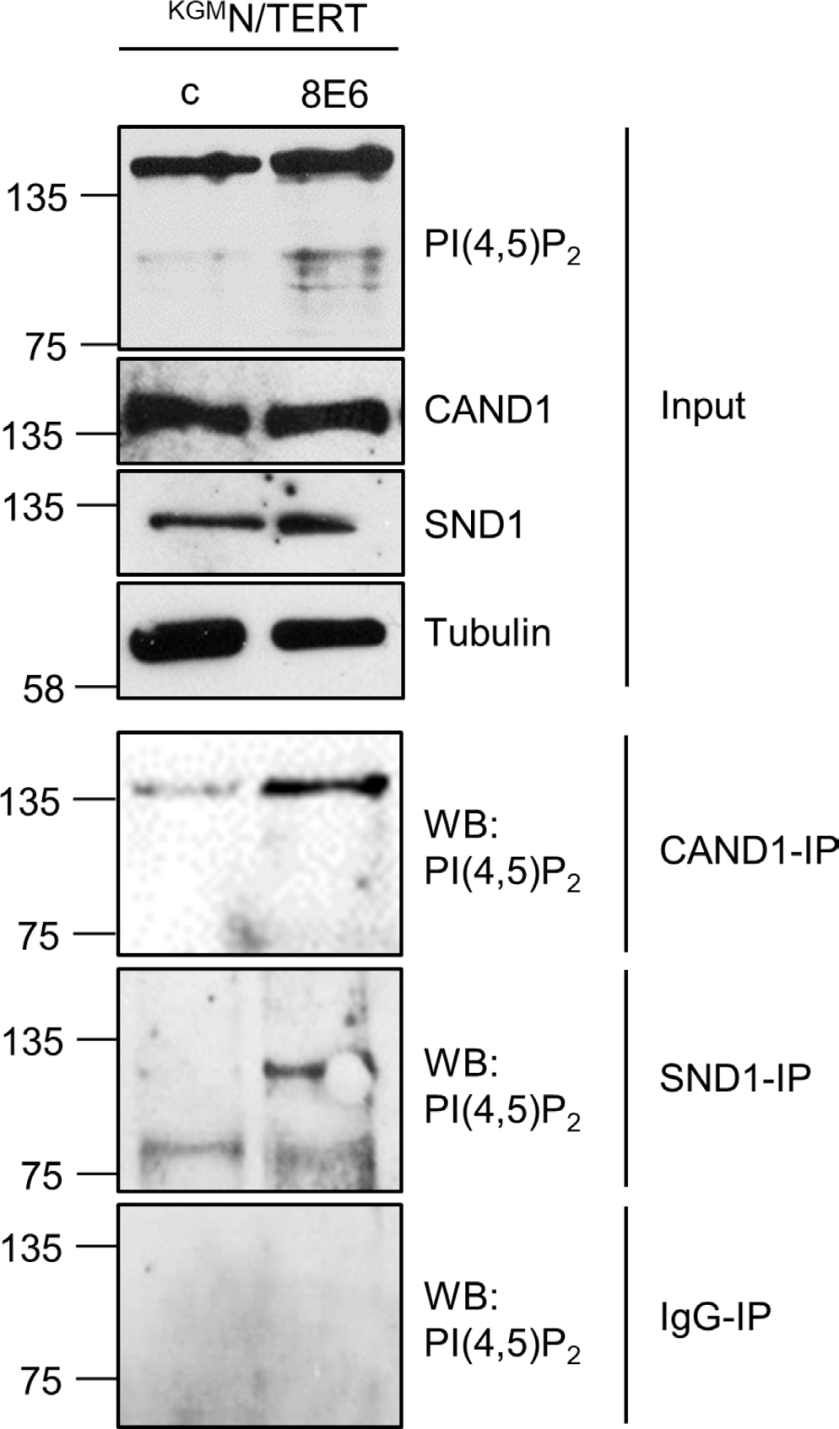
Identification of CAND1 and SND1 as PI(4,5)P_2_ binding cellular proteins Extracts from ^KGM^N/TERT-pLXSN and ^KGM^N/TERT-8E6 were incubated with A/G-agarose coupled with either CAND1, SND1 or normal IgG-mouse antibodies. Co-immunopecipitated PI(4,5)P_2_ and 10% of the input extracts were detected by Western blot with specific antibodies against PI(4,5)P_2_. Total levels of PI(4,5)P_2_, CAND1 and SND1 were detected using specific antibodies. Equal protein loading was confirmed by immunoblotting for tubulin.

In order to identify the PI(4,5)P_2_ bound proteins in ^KGM^N/TERT-8E6 cells we performed Co-IP experiments using the anti-PI(4,5)P_2_ antibody with subsequent mass spectrometric analysis. The comparative proteomic profiling of total E6 cell extracts compared with wild-type keratinocytes revealed 10 nuclear proteins with a molecular mass between 75–150 kDa by at least 2 unique peptides in both control and E6 positive cells (Supplementary Table 1). To verify these findings, both Co-IP and siRNA-knockdown experiments were carried out, which identified CAND1 (Cullin-associated NEDD8-dissociated protein-1) and SND1 (Staphylococcal nuclease domain-containing protein 1) as nuclear proteins differentially phospholipidized in the presence of E6. While total levels of CAND1 and SND1 were not significantly changed, immunoprecipitates of CAND1 and SND1 were found to be bound to PI(4,5)P_2_ in ^KGM^N/TERT-8E6, but not ^KGM^N/TERT-pLXSN cells (Figure 8).

## DISCUSSION

Phosphoinositides have proven to be key lipid messengers regulating almost every singular aspect of eukaryotic cell physiology [36]. Among them, PI(4,5)P_2_ has been demonstrated to play a key role in generating other phosphoinositide species as well as other lipid messengers [37]. PI(4,5)P_2_ directly interacts with a vast range of proteins called PI(4,5)P_2_ effectors. Its concentration is dramatically altered by external or internal stimuli to efficiently regulate intracellular functions [25, 26], a mechanism we have now proven to be hijacked by the HPV8-E6 oncoprotein. Furthermore, nuclear, non-membrane pools of signaling phosphoinositides have previously been shown to be crucial for carcinogenesis [18]. Our initial observation, that PI(4,5)P_2_ associated protein bands were found in ^KGM^N/TERTs comparing them to ^RM+^N/TERTs, combined with a decrease of PI(4,5)P_2_ signals following calcium induced differentiation of ^KGM^N/TERTs provides even more compelling evidence that accumulation of PI(4,5)P_2_ is taking place in non-differentiated keratinocytes. The fact that PI(4,5)P_2_ bound protein signals and total levels of phosphoinositides were observed to be enhanced in the presence of HPV8-E6 further underpins the potential role of PI(4,5)P_2_ in maintenance of an undifferentiated cell phenotype. The shift towards polyunsaturated lipid species 38:4 and 38:3, which are mainly comprised of 18:0/20:4 and 18:0/20:3, further suggests that these lipids may contribute to HPV8-E6 induced cell transformation.

The highly significant PI(4,5)P_2_ signals in skin hyperplasia following UV treatment in wt mice and K14-HPV8-E6 transgenic mice, combined with the observation of a reduction of PI(4,5)P_2_ to undetectable levels upon skin healing in wt mouse. Satz unvollständig, support a crucial role of PI(4,5)P_2_ in epidermal wound healing, and re-establishment of skin homeostasis following UV irradiation. Activated keratinocytes driving re-epithelialization may fail to switch off their activated status upon wound-healing in HPV infected skin [4]. Collectively, this may result in constantly elevated nuclear PI(4,5)P_2_ levels, with profound promoting effects on both initiation and growth of HPV positive skin tumors. The nuclear staining pattern of PI(4,5)P_2_ in EV skin lesions (with high betaPV loads) and absence of PI(4,5)P_2_ in SCC with low betaPV loads, basal cell carcinoma, melanoma or Merkel cell carcinoma may imply a role of PI(4,5)P_2_ signaling in betaPV dependent skin carcinogenesis. The observation that high betaPV loads correlated with high PI(4,5)P_2_ levels underpins the importance of PI(4,5)P_2_ in betaPV mediated skin SCC development.

As the E6 mutant W61A/W63A was unable to form a complex with MAML1 but still led to an increase of PI(4,5)P_2_, we provide evidence that an E6 mediated increase of PI(4,5)P_2_ is independent of its ability to interfere with the MAML1/NOTCH signaling pathway. In a recent study, seeking to define key amino acids in E6 relevant for MAML1 binding, the authors identified the residue K64 as also being crucial for MAML1 binding, indicating the importance of amino acids around position 61–64 [11]. Taken together, the elevated levels of nuclear PI(4,5)P_2_ in HPV8 and HPV16 positive patient tissues suggest a high significance of our findings for keratinocyte cancer development and may thus be relevant for novel cancer therapy concepts by targeting nuclear PI(4,5)P_2_-driven tumor growth.

We next asked, which PI(4,5)P_2_ metabolic enzymes may contribute to PI(4,5)P_2_ accumulation. Although PIP4KII and PIP5KI synthesize the same end product, they are not functionally redundant [38]. Since the cellular levels of PI4P are much higher than those of PI5P, it seems likely that the majority of nuclear PI(4,5)P_2_ is synthesized through PIP5KI [34]. The increase of PI(4,5)P_2_ signals in ^KGM^N/TERT-8E6 cells is associated with an increase in gene expression of these kinase isoforms. Combined with the fact that knockdown of a single isoform did not affect PI(4,5)P_2_ levels led to the overall conclusion that several kinases are simultaneously contributing to nuclear PI(4,5)P_2_ production. Whether E6 binding to OCRL1 may inactivate its phosphatase function and thereby contributes to the persistent high levels of PI(4,5)P_2_ needs to be determined in future experiments. In addition, we could also show that HPV8-E6 binds PI, which suggests the intriguing possibility that HPV8-E6 may contain a phosphoinositide-binding domain. The interaction of E6 with PI may enforce phosphorylation of PI leading to the generation of the PI(4,5)P_2_ precursors PI4P and PI5P. We therefore conclude, that all three processes could be relevant mechanisms involved in PI(4,5)P_2_ metabolism.

Deregulation of the phosphoinositide signaling axis is associated with cancer development [39]. To assess possible consequences of the PI(4,5)P_2_ metabolic pathway in betaPV associated skin cancer development, it should be noted that UV radiation - the major risk factor for skin cancer - usually causes PI(4,5)P_2_ depletion during apoptosis independently of and prior to caspase activation [40, 41]. In addition, downregulation of PIP4KII and subsequent reduction of PI(4,5)P_2_ triggers apoptosis in MDA-MB-231 breast cancer and Cos7 cells [42]. Regarding the co-factorial role of high betaPV loads in skin cancer development, the virus may counteract these pro-apoptotic signals through upregulation of PI(4,5)P_2_.

Until now, only few nuclear PI(4,5)P_2_ effector proteins have been identified and their functional classification pointed to roles in mRNA transcription regulation, mRNA splicing and protein folding [21]. By screening for potential PI(4,5)P_2_ phospholipidized nuclear proteins in HPV8-E6 positive cells, the multifunctional regulator proteins CAND1 and SND1, which are known to be direct interaction partners [43, 44] were identified. CAND1 is a F-box exchange factor required for loading substrates onto the Cullin-Ring-ubiquitin ligase complex required for ubiquitination and proteasomal degradation of numerous protein substrates [45]. SND1 is known to be critically involved in virtually all pathways of gene expression, ranging from transcription to RNA silencing exerting a biochemical function as both a scaffolding molecule and/or a nuclease. SND1 is indispensable for normal development and stress resistance, whereas its deregulation is closely associated with various types of cancer [46, 47]. SND1 is higher expressed in basal as well as hair follicle keratinocytes than in terminally differentiated cells of the human interfollicular epidermis [48]. HPV induced phospholipidation of SND1 in keratinocytes may therefore contribute to HPV-mediated skin tumorigenesis. Since CAND1 and SND1 total protein levels were not changed upon HPV8-E6 expression, the observed rise in CAND1- and SND1-associated PI(4,5)P_2_ signals may result from enhanced phospholipidation. Our findings suggest that the CAND1-SND1 pathway may be an important regulatory node for HPV early gene functions, which will be subjects of further mechanistical studies.

In conclusion, we identified the PI(4,5)P_2_ metabolism as a novel target of oncogenic HPV induced carcinogenesis. Further studies will be needed to expand our understanding of the exact biological and oncogenic implications, which may pave the way for entirely novel scientific avenues in the fields of HPV-mediated carcinogenesis.

## MATERIALS AND METHODS

### Cell lines and treatments

Isogenic N/TERTs (kindly provided by James Rheinwald, Harvard Medical School, Boston, MA, USA) [29] were either cultivated in KGM-Gold (^KGM^N/TERTs, Lonza, Cologne, Germany; containing low (0.05 mM) Calcium) or in RM+ media (^RM+^N/TERTs) [49]. Differentiation of ^KGM^N/TERTs was accomplished by treatment with 2 mM CaCl_2_ for up to 8 days. Retroviral transduction of N/TERTs was performed using the retroviral vector pLXSN-HPV8-E6 as previously described [50]. The generation of organotypic skin cultures of HPV8-E6 expressing PHK has been described elsewhere [51]. The HPV negative cervical carcinoma cell line C33a (purchased from American type Culture Collection (ATCC #HTB-31)) was grown in Dulbecco’s modified Eagle medium (DMEM, Life Technologies, Darmstadt, Germany), supplemented with 10% fetal calf serum (FCS).

For the protein-lipid overlay assay on PIP-Strips (Echelon Biosciences), C33a cells were transiently transfected in 6-wells by the CaCl_2_-method with the empty eukaryotic expression vector pXJ41-Flag or pXJ41-8E6-Flag (kindly provided by Dr. G. Steger, Institute of Virology, University of Cologne, Germany) coding for Flag-tagged HPV8-E6. Total cell extracts were generated 48 h post transfection using low salt dilution buffer (LSDB) (50 mM Tris/HCl (pH 8), 20 % Glycerol, 100 mM KCl, 0,1% NP40, 1 mM DTT, 50 mM NaF, 1 mM orthovanadate, 1 mM PMSF, supplemented with 1× Cocktail Protease Inhibitors (Roche Diagnostics, Mannheim, Germany)). PIP-Strips were developed by incubation with cell extracts and an anti-Flag antibody. N/TERTs and C33a cells were authenticated by the Leibniz-Institute (DSMZ-German Collection of Microorganisms and Cell Cultures).

### Western blotting and co-immunoprecipitation

For Western blot analysis, cells were trypsinized, pelleted by centrifugation and lysed on ice in LSDB buffer. The extracts were sonicated, and protein concentrations were determined using the Bio-Rad Protein Assay (Bio-Rad, Munich, Germany). Cell extracts were resolved by SDS-PAGE and transferred to a nitrocellulose membrane. After the membrane had been blocked with 5 % skimmed milk in TBST (10 mM Tris/HCl (pH 8.0), 150 mM NaCl, 0.05 % Tween 20) for 1h, the blots were probed with antibodies to Loricrin (ab24722, Abcam, Cambridge, UK), PI(4,5)P_2_ (clone 2C11, Santa Cruz, Heidelberg, Germany) [30] and tubulin (clone YL1/2, Abcam), which was used as loading control. Immunoreactive proteins were visualized using horse-reddish-peroxidase coupled secondary antibodies and BM Chemiluminescence Blotting Substrate (Roche Diagnostic). For Co-IP experiments, cell extracts of transiently transfected C33a were incubated with antibody coupled agarose for 2 h at 4° C, followed by three washes with LSDB containing different KCl concentrations. Co-precipitated cellular proteins were detected by Western blotting. The antibodies used for these studies were: anti-HA (3F10, Roche Diagnostics), anti-Flag (clone M5, Sigma), anti-OCRL-1 (8797, Cell Signaling), anti-MAML1 (D3K7B, Cell Signaling), anti-CAND1 (G-3, Santa Cruz), anti-SND1 (F-5, Santa Cruz).

### Quantitative PCR following reverse transcription (RT-qPCR)

Total RNA was isolated from cells using the RNeasy kit, and DNase digestion was performed on a column using RNase-free DNase according to the manufacturer’s instructions (Qiagen, Hilden, Germany). One microgram of total RNA was reverse transcribed using the Omniscript RT kit (Qiagen) with 10 μM random nonamers (TIB MOLBIOL, Berlin, Germany) and 1 μM oligo(dT_23_) primer (Sigma), as well as 10 units of RNase inhibitor (Fermentas, St. Leon-Rot, Germany). Quantitative PCR (qPCR) was performed using the Light-Cycler system (Roche). Amplified PCR fragments were cloned into pJET1.2 (Qiagen) to generate absolute standards with primers also used for subsequent qPCR analysis. Samples were analyzed in duplicate together with a 10-fold dilution series of standard plasmids. The mRNA expression levels of target genes were normalized to the mRNA levels of hypoxanthine phosphoribosyltransferase 1 (HPRT1). The primers used in this study had the following 5′-to-3′ sequences:

HPRT1-fw: CCTAAGATGAGCGCAAGTTGAA,

HPRT1-bw: CCACAGGACTAGAACACCTGCTAA;

PIP4KIIα-fw: ATGGAATTAAGTGCCATGAAAAC

PIP4KIIα-bw: GCATCATAATGAGTAAGGATGTCAAT

PIP4KIIβ-fw: TGCATGTGGGAGAGGAGAGT

PIP4KIIβ-bw: TCAGCTGTGCCAAGAACTCA

PIP4KIIγ-fw: GTGTTCCTGTGGGGCGTA

PIP4KIIγ-bw: TGATCTTGGAGCTGGCCTTA

PIP5KIα-fw: CCAACATAAAGAGGCGGAAT

PIP5KIα-bw: AGGGTTCTGGTTGAGGTTCAT

PIP5KIβ-fw: AGCAGCCTTGATGAAGAAGC

PIP5KIβ-bw: GAAGAAGATGAAATTGTGGTTGC

PIP5KIγ-fw: AAGGAGGCCGAGTTCCTG

PIP5KIγ-bw: CGGGTTCTGGTTGAGGTTC.

### Ethics statement

The generation of K14-HPV8-E6 transgenic mice (FVB/n genetic background, Charles River Laboratories, Sulzfeld, Germany) and description of the UV irradiation protocol were described previously [12, 52]. The generation of the transgenic mice and the UV irradiation protocols were approved by the governmental animal care office North-Rhine-Westphalia (protocol no. 87-51.04.2010.A203) and were in accordance with the German Animal Welfare Act as well as the German Regulation for the protection of animals used for experimental purposes. EV skin lesions were removed during routine surgical excisions from which paraffin blocks were generated. The collection and analysis of EV skin was approved by the local ethics-committee at the Medical University of Warsaw, Poland. Tissue-micro-array (TMA) specimens of formalin-fixed, paraffin-embedded HPV16 positive CIN lesions (CINI-III and CC from individual patients) were obtained from the archive files of the Department of Pathology, University of Cologne. Clinical information was obtained from the patients’ medical records. The collection of CIN lesions was realized according to BioMaSOTA votum (approval number 13-091) at the University of Cologne, Germany. Written informed consent, which was conducted in accordance with the Declaration of Helsinki, was obtained for the use of patient foreskin for isolation of primary keratinocytes. For biopsy materials from archival paraffin blocks of human Merkel cell carcinoma, basal cell carcinoma and malignant melanoma informed consent was obtained from all the subjects and ethical approval obtained from the Ethics Committee at the University of Cologne.

### Immunofluorescence staining of skin samples

Four-micrometer formalin-fixed, paraffin-embedded sections were de-paraffinized with xylene. Samples were hydrated through a descending alcohol series and endogenous peroxidases were inactivated by incubation in 3% H_2_O_2_ in methanol for 20 min. Antigen unmasking was performed by boiling the tissue sections in 10 mM citric buffer (pH6; for PI(4,5)P_2_ and H3K4me3 detection) for 3 min in a microwave followed by 15 min resting at RT. Blocking of unspecific antigen sites was achieved with 50% goat serum (Thermo Scientific, Schwerte, Germany) in PBS for 1h at RT. Incubation with primary antibody against PI(4,5)P_2_ was done in a dilution of 1:250 in 2% goat serum over night at 4° C. Detection of PI(4,5)P_2_ antibodies was achieved by incubating the sections with a HRP-conjugated goat-anti-mouse-IgM antibody (Santa Cruz) diluted 1:1,000 in 2% goat serum for 1h at RT. The fluorescence signals were generated with the ‘‘TSA™, Fluorescein System’’ (Perkin Elmer, Rodgau, Germany). After the sections were counterstained with 4′,6-diamidino-2-phenylindole (DAPI) and embedded in Immunomount (Fisher Scientific, Schwerte) the specific signals were visualized by immunofluorescence microscopy using the Leica microscope DMI6000B, Leica camera DFC365FX and Leica Application Suite software v3.3.0.16799.

### Phosphoinositide extraction and quantification

Phosphoinositide extraction was performed as described in [53] with a few modifications. Briefly, cell pellets were subjected to a two step neutral-acidic lipid extraction in the presence of PI(4)P 17:0/20:4 and PI(4,5)P_2_ 17:0/20:4 (Avanti Polar Lipids). Pellets were resuspended in 1ml chloroform/methanol (1:2, v/v) and incubated for 10 min at RT. The sample was vortexed for 30s every 3 min. After precipitation (13,800 × g, 2 min at 4° C), the supernatant was transferred to a new Eppendorf tube. For acidic extraction, the remaining pellet was resuspended in 750 μl chloroform/methanol/37%HCl (40:80:1,v/v/v) and incubated for 15 min at RT while vortexing the sample for 30 s every 5 min. After transferring the tube to ice, 250 μl cold chloroform and 450 μl cold 0.1M HCl was added followed by 1 min vortexing and centrifugation (6,500 × g, 2 min at 4° C). The bottom organic phase was transferred to a new tube. An aliquot of the organic phase was adjusted to 10 mM ammonium acetate in methanol and analyzed by mass spectrometric analysis on a QTRAP6500 (Sciex) equipped with CHIP-based infusion (Nanomate, Advion). Phosphoinositides were analyzed by positive ion mode neutral loss scanning selecting for fragment ions with m/z 375 phosphatidylinositol phosphate (PIP) and m/z 437 phosphatidylinositol-bisphosphate (PIP_2_). Lipid quantification was done using LipidView software v1.2 by SCIEX.

### Proteomic analysis

Proteomic analysis was performed as recently described [54]. Briefly, proteins immunoprecipitated using mouse anti-PI(4,5)P_2_ IgM antibodies were reduced by dithiothreitol (100 mM) and alkylated (550 mM IAA) prior to digestion with trypsin and Lys-C overnight at 37° C. Generated peptides were extracted by incubation with increasing amounts of acetonitrile, concentrated in a speed-vac and primed prior to liquid chromatography and tandem mass spectrometry (LC-MS/MS) analysis by the STAGE tip technique [55]. For LC-MS/MS an easy nLC 1000 (Thermo Scientific) was coupled to the quadrupole-based Q Exactive Plus (Thermo Scientific) instrument by a nano-spray ionization source. Peptides were separated on a 50 cm in-house-packed column by a two-solvent buffer system: buffer A (0.1% formic acid) and B (0.1% formic acid in acetonitrile). The content of buffer B was increased from 7% to 23% within 40 min and followed by an increase to 45% in 5 min and a washing and re-equilibration step before the next sample injection. The mass spectrometer operated in a Top 10 data-dependent mode using the following settings: MS1: 70.000 (at 200 m = z) resolution, 3e6 AGC target, 20ms maximum injection time, 300–1.750 scan range; MS2: 35.000 (at 200 m = z) resolution, 5e5 AGC target, 108ms maximum injection time, 1.8 m/z isolation window, 27 normalized collision energy. Bioinformatic analysis of all raw files was processed by MaxQuant 1.5.1.2 and Perseus 1.5.0.3.1.

### Statistical analysis

All experiments were repeated a minimum of three times. All data from RT-qPCRs were expressed as mean ± SEM. Results of the lipid analyses are presented as mean ± SD. The data presented as immunoblots or images of immunofluorescence analysis are from a representative experiment, which was qualitatively similar in the replicate experiments. Statistical significance was determined with unpaired 2-tailed Student’s *t*-test. The asterisks shown in the figures indicate significant differences of experimental groups (^*^p < 0.05; ^**^p < 0.01, ^***^p < 0.001).

## SUPPLEMENTARY MATERIALS FIGURE AND TABLE



## Abbreviations

HPV: human papillomavirus
SCC: squamous cell carcinoma
EV: Epidermodysplasia verruciformis
PHK: primary human keratinocyte
UV: ultra-violet
PI(4,5)P_2_: phosphatidylinositol-4,5-bisphosphate
PIP_2_: phosphatidylinositol-bisphosphate
PI: phosphatidy linositol
PI4P: Phosphatidylinositol-4-phosphate
PI5P: Phosphatidylinositol-5-phosphate
PIP5KI: phosphatidy linositol-4-phosphate-5-kinase type I
PIP4KII: phosphatidy linositol-5-phosphate 4-kinase type II
MAML1: Mastermind-like protein 1
RT-qPCR: reverse transcription quantitative PCR
