# Supplementary material for: Phospholipidation of nuclear proteins by the human papillomavirus E6 oncoprotein: implication in carcinogenesis
Source: Oncotarget. 2018 Sep 25;9(75):34142–58. doi: 10.18632/oncotarget.26140 (PMC6183346; doi:10.18632/oncotarget.26140)
Supplement: Supplementary file 1 [file oncotarget-09-34142-s001.pdf]

Phospholipidation of nuclear proteins by the human papillomavirus E6 oncoprotein: implication in carcinogenesis

SUPPLEMENTARY MATERIALS

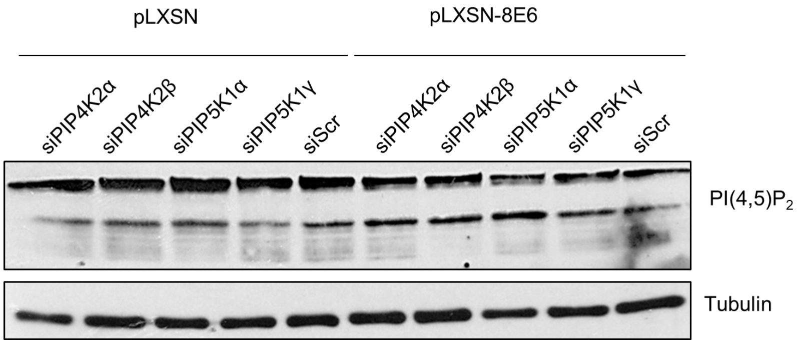

Supplementary Figure 1: Representative Western blots for PI(4,5)P_2 in total cell extracts of ^{KGM}N/TERT-pLXSN and ^{KGM}N/TERT-8E6 cells transfected with siRNA against PIP4KIIα, β and PIP5KIα, γ. Equal protein loading was confirmed by immunoblotting for tubulin. Here, the liposome based transfection method did consistently (*n* = 4 independent experiments) affect the pLXSN-8E6 mediated effect of PI(4,5)P_2 phospholipidation of cellular proteins.

Supplementary Table 1: Nuclear proteins with a molecular mass between 75–150 kDa are identified by PI(4,5)P_2 Co-IP and subsequent mass spectrometric analysis

Protein	Gene	kDa	References for known interactors
ATP-dependent RNA helicase A	DHX9	140	Catimel *et al*. 2008 [1]
Cullin-associated NEDD8-dissociated protein 1	CAND1	136	Catimel *et al*. 2008 [1]
Importin-7	IPO7	119	Catimel *et al*. 2008 [1]
Importin-5	IPO5	109	
Ankycorbin (nuclear)	RAI14	109	Catimel *et al*. 2008 [1]
26S proteasome non-ATPase regulatory subunit 1	PSMD1	105	
Protein unc-45 homolog A	UNC45A	103	
Staphylococcal nuclease domain-containing protein 1	SND1	102	Lewis *et al*. 2011 [2]
Transcription intermediary factor 1-beta	TRIM28	88	
Nucleolar RNA helicase 2	DDX21	87	Lewis *et al*., 2011 [2]

References for known interactors are given.

REFERENCES

1. Catimel B, Schieber C, Condron M, Patsiouras H, Connolly L, Catimel J, Nice EC, Burgess AW, Holmes AB. The PI(3,5)P_2 and PI(4,5)P_2 interactomes. J Proteome Res. 2008; 7:5295–313.
2. Lewis AE, Sommer L, Arntzen MØ, Strahm Y, Morrice NA, Divecha N, D'Santos CS. Identification of nuclear phosphatidylinositol 4,5-bisphosphate-interacting proteins by neomycin extraction. Mol Cell Proteomics. 2011; 10:M110.003376.
